# Dental management of patients with renal diseases or undergoing renal transplant

**DOI:** 10.1016/j.sdentj.2023.11.023

**Published:** 2024-01-09

**Authors:** Hamad Albagieh, Asim Alosimi, Abdulrahman Aldhuhayan, Abdulrahman AlAbdulkarim, Bader Fatani, Abdulaziz Alabood

**Affiliations:** a Oral Medicine and Diagnostic Sciences Department, College of Dentistry, King Saud University, Riyadh, Saudi Arabia; b College of Dentistry, King Saud University, Riyadh, Saudi Arabia

**Keywords:** Renal disease, Renal transplant, Dental consideration, Oral manifestation, Treatment plan modification, Risk of bleeding and infection, Drug-drug interaction, Emergency

## Abstract

Background: The kidneys carry out many essential processes. A reduced glomerular filtration rate is the primary cause of renal failure. Patients with chronic kidney disease are significantly increasing all around the world. Patients with varying degrees of renal disease will be seen on the dental chair, and the dentist must be aware of dental considerations when treating such patient. Dental clinicians should carefully evaluate the oral findings and general condition of the renal disease patient. Objective: To increase the awareness of dentists in the dental management of patients with renal diseases or undergoing renal transplant. Methods: This study reviews 56 articles searched on two databases PubMed and Google Scholar with English language. Conclusion: Every dentist should be well-informed regarding dental considerations and oral manifestations when treating a patient with any renal disease. Simple treatment plan modification can prevent the evolution of mild to critical medical or dental complications in renal disease patients.

## 1 Introduction

Kidneys are essential in balancing body fluid volumes and the composition of the extracellular and intracellular fluid compartments (Olivas-Escárcega et al., 2008). Renal disease and renal transplants have highly grown in the last decade due to the loss of kidney function to excrete waste from the blood and the inability to regulate electrolytes (Ferguson and Whyman, 1998, Gudapati et al., 2002, Sharma and Pradeep, 2007, Proctor et al., 2005). Renal disease frequently has oral symptoms which can manifest as distinct symptoms of multisystem diseases that impact the kidneys (such as vasculitis) or as typical oral pathologies more prevalent in people with end-stage renal disease (Summers et al., 2007). Even though patients on kidney transplant treatment have higher oral and dental diseases, dental clinic visits are infrequent, and dentists are generally unaware of the issue (Summers et al., 2007). The earliest sign of dental finding in these patients is typically the presence of enamel hypoplasia due to severe metabolic disturbance throughout the mineralization process (Lucas & Roberts, 2005). Patients with chronic kidney disease are significantly increasing all around the world (Yuan et al., 2017). In the Kingdom of Saudi Arabia, there are over 20,000 patients on dialysis and 9,810 patients undergoing follow-up after kidney transplantation, and the combined prevalence of renal replacement therapy is estimated to be 294.3 per million population (Mousa et al., 2021). All these numbers indicate that chronic kidney disease is a significant public health concern in the Kingdom of Saudi Arabia (Mousa et al., 2021).

## 2 Methods

This study reviews 56 articles from a search on two databases (PubMed:1960 to 2022 and Google Scholar: 1960 to 2022), according to the following search criteria: ‘Dental AND (renal failure OR kidney disease OR haemodialysis OR transplantation)’ and in the English language. We also manually checked the reference list of related review articles and editorials to identify additional studies. The full texts of studies were retrieved and evaluated for quality assessment and data synthesis. The summary data were extracted from enrolled studies. The only exclusion criterion was whether the article topic was unrelated to the research question.

## 3 Discussion

### 3.1 Renal disease

The kidneys carry out many essential processes. The reduced glomerular filtration rate (GFR) is the primary cause of renal failure (Singh et al., 2014). The results include osteodystrophy, elevated blood pressure, weight loss, anemia, and neuropathy. The renal condition with the most significant dental implications is chronic renal disease (CRD) (Singh et al., 2014), a clinical illness that worsens over time and is characterized by a decline in the kidney's ability to maintain homeostasis and excrete waste products. In various Western nations, there are roughly 200 instances of CRF per million people (Akar et al., 2011, Almeida et al., 2016, Parsegian et al., 2022, Sulejmanagić et al., 2005). The National Kidney Foundation classifies chronic kidney disease into five stages based on the glomerular filtration rate (GFR) (National Kidney Foundation, 2015). Stage 1 is considered asymptomatic and known by a normal or slight decline in renal function (10 ml/min/1.73 m2) and may come with very minimal kidney damage; stage 2 is characterized by a mild decrease in the GFR (11 – 40 ml/min/1.73 m2), In stage 3a the GFR is decreased by (41–55 ml/min/1.73 m2) where in stage 3b the GFR is decreased by (56–70 ml/min/1.73 m2). Stage 4 is characterized by a severe decrease in GFR (71–85 ml/min/1.73 m2) and stage 5 is considered kidney failure with a decrease in GFR by more than 85 and patients in this stage are on dialysis (Little et al., 2018, National Kidney Foundation, 2015). End-stage renal disease is the final condition following numerous primary renal and systemic diseases, which in turn cause kidney function loss (De la Rosa García et al., 2006).

### 3.2 Causes of chronic renal disease

• Glomerulonephritis (25 % of cases).
• Diabetes mellitus.
• Severe pyelonephritis.
• Hypertension
• Polycystic kidney disease.
• Drugs, nonsteroidal anti-inflammatory drugs (NSAIDs).
• Connective tissue diseases such as systemic lupus erythematosus and polyarteritis nodosa.
• Renal stones. (Fitzpatrick et al., 2008).

### 3.3 Renal transplant

Patients with end-stage renal disease should consider kidney transplantation as it is the best line of treatment for patients with end-stage renal failure (Al-Mohaya et al., 2009, Levarda-Hudolin, 2016, Wilczyńska-Borawska et al., 2010). Renal transplantation can improve the quality and longevity of life for many patients. Nevertheless, the use of immunosuppressant medication for the treatment of renal transplant has many side effects, including cardiovascular risk, cancer, and infections that could be life-threatening (López-Pintor et al., 2011). Renal allografts may be cadaveric or from living donors, either related or non-related (although those from living relatives give rise to the best prognosis). Cadaveric organs are allocated based on HLA tissue typing, ABO compatibility, and the age and size of the donor and recipient (Almeida et al., 2016).

### 3.4 Dental consideration

In every dental clinic, patients with varying degrees of renal disease will be seen on the dental chair, and the dentist must be aware of dental considerations when treating such patient (Klassen and Krasko, 2002, Proctor et al., 2005). Patients with early renal disease can be given amide local anesthesia as it is mainly metabolized in plasma, and these patients are not contraindicated for dental treatment. Many dental treatments can be performed successfully (Sulejmanagić et al., 2005). However, it is important to treat any odontogenic infections, especially in renal transplant patients; if these infections are left untreated, it will increase the chance of kidney rejection, which could lead to morbidity (Akar et al., 2011). It is recommended to give prophylactic antibiotics one hour before the dental procedure; however, it is essential to refer the patient to a renal specialist who can provide the dentist with information concerning the state of the disease and the adopted therapeutic measures (Georgakopoulou et al., 2011, Levy, h. m. , 1988). The renal specialist must have previously agreed upon any changes in drugs routinely used by the patient or other aspects of treatment (Georgakopoulou et al., 2011). Nephrologists have agreed to use NSAID as an analgesic, in addition to mouth-rinse with an alcohol-free mouthwash that is used to minimize oral dryness (Akar et al., 2011, Levy, h. m. , 1988). Patients with renal disease are at high risk of caries formation due to a high carbohydrate diet (thus, it is important to reduce renal workload), poor oral hygiene, low saliva flow, or a combination of more than one factor (Gupta et al., 2015, Saif et al., 2011a, Saif et al., 2011b). A wide range of conditions that affect the hard or soft tissues of the mouth is linked to renal failure (Dioguardi et al., 2015, Summers et al., 2007). Individuals with renal impairment are more likely to develop periodontal disease, and patients receiving both hemodialysis and peritoneal dialysis are more likely to have poor oral health (Summers et al., 2007). Patients on dialysis have been reported to have bone lesions, xerostomia, unpleasant taste, delayed tooth eruption, and calcifications leading to obliteration of the pulp chamber and canals (Craig, 2007). In addition to enamel hypoplasia, foul odor, oral hairy leukoplakia, uremic stomatitis, oral malignancy, and gingival hypertrophy are also reported (Dioguardi et al., 2015). Immunosuppressants and antihypertensives increase the risk of gingival hyperplasia in patients who have had kidney transplants (Dioguardi et al., 2015, Summers et al., 2007). Oral lesions, whether infectious or neoplastic, are common in these people (Summers et al., 2007). In approximately 50 % of patients, extra-oral manifestations included pedal edema, pale skin, swollen face, vomiting, bruises on the skin, and nausea (Nandan et al., 2005).

### 3.5 **Oral manifestation (Mucosa and Skeletal)** patients with renal disease can present with a wide range of oral manifestations, which include the following

#### 3.5.1 Amelogenesis imperfecta

Amelogenesis imperfecta is a hereditary condition characterized by a reduced enamel thickness with normal hardness and is classified as hypoplastic amelogenesis imperfecta, while discoloration with normal thickness and decreased hardness is known as hypermineralized amelogenesis imperfecta (Misgar et al., 2017, Saif et al., 2011a, Saif et al., 2011b). There are rare reports of it being linked to metabolic diseases and multi-organ syndromes, A number of metabolic disorders have been linked to amelogenesis imperfecta, such as hypocalciuria and distal renal tubular acidosis (Misgar et al., 2017). Enamel-renal syndrome is a term used to describe amelogenesis imperfecta with nephrocalcinosis. This is a rare syndrome and certain cases of it can lead to renal insufficiency (Misgar et al., 2017).

#### 3.5.2 Pallor of the oral mucosa

Minerals and vitamins are essential for keeping healthy mucous membranes. The oral cavity may show early symptoms of nutritional deficits (Thomas and Mirowski, 2010). Patients with chronic renal failure experience pallor in the oral mucosa related to anemia, which may be caused by the kidneys producing less erythropoietin, renal loss of red blood cells, marrow fibrosis, and increased red cell fragility with early destruction (Patil et al., 2012, Saif et al., 2011a, Saif et al., 2011b). The pallor of oral mucosa was reported to be (87 %) in chronic renal failure patients (Patil et al., 2012, Saif et al., 2011a, Saif et al., 2011b).

#### 3.5.3 Periodontal diseases

Periodontal diseases are common among patients with renal failure, most often caused by Gram-negative anaerobic bacteria that colonize supragingival and subgingival plaques and produce local and systemic immunological and inflammatory responses (Nadeem et al., 2009). Periodontal diseases include recession, loss of attachment, deep pocket, gingival overgrowth, plaque accumulation, and periodontitis (Davidovich et al., 2005, Singh and Pal;Mittal, Sanjeev;Garg, Sushant;Chandni. , 2014). Altered calcium and phosphate level may lead to changes in alveolar bone like lamina dura loss. However, immunosuppression decreases the immune system's response to periodontal infections (Davidovich et al., 2005). Halitosis was reported to be 34 %in chronic renal failure patients (Patil et al., 2012).

#### 3.5.4 Xerostomia

Xerostomia is a sensation of dryness in the mouth and is caused by a decrease in salivary flow. Side effects of drugs or restriction of fluid intake are important to accommodate the reduced excretory fluid intake (Dirschnabel et al., 2011). Dry mouth is common and significant among dialysis patients, and the geographic tongue is more common in kidney transplant patients. Chronic xerostomia predisposes patients to sialadenitis, caries, oral inflammation, infection, and sore mouth; also, it can alter the taste sensation and odor where the patient complains of metallic taste and unpleasant ammonia-like oral odor; metallic taste is more predominant in renal dialysis patients (Dirschnabel et al., 2011). Those who complain of dry mouth need to get regular dental examinations. Chewing gum and proper oral hygiene are two possible treatments for xerostomia (Gupta et al., 2015, Proctor et al., 2005, Summers et al., 2007).

#### 3.5.5 Candidiasis

Candida albicans are opportunistic pathogens that usually live in the oral cavity in a latent state (Olivas-Escárcega et al., 2008). Predisposing factors are frequently connected to the transformation of Candida from a commensal to a pathogen (Lopez-Pintor et al., 2013). The systemic factors promoting oral candidiasis in renal transplant patients are immunosuppressant dose, diabetes mellitus, prolonged antibiotic use, leukopenia, and old age. Local factors, such as poor oral hygiene and dental and oral health conditions, might change the mucosal barrier or reduce the quality or quantity of saliva which can contribute to oral candidiasis (Lopez-Pintor et al., 2013). However, an extreme increase in the incidence of oral candidiasis has been observed in immunocompromised patients (Olivas-Escárcega et al., 2008). Such individuals' oral candidiasis increases their risk of developing esophageal candidiasis, an invasive form with high morbidity. In addition, oral candidiasis is more severe in the immediate post transplantation period (Lopez-Pintor et al., 2013).

#### 3.5.6 Uremic stomatitis

This is a rare mucosal condition and is seen in patients with advanced or chronic renal failure (Sudarshan et al., 2012, Summers et al., 2007). Any area of the oral mucosa can develop and is characterized by the presence of lesions with an erythema; these lesions are coated with pseudo-membranous exudates that can be removed, leaving an intact or ulcerated mucosa (Patil et al., 2012). The conversion of salivary urea by bacterial ureases to ammonia, which results in a “chemical burn,” has been proposed as the source of these frequently painful lesions. Mouthwashes with antibacterial agents may reduce these symptoms (Sudarshan et al., 2012, Summers et al., 2007).

#### 3.5.7 Bone abnormalities

It is well-known that dialysis patients experience early jawbone loss, which can result in mandibular and maxillary fractures (Yuan et al., 2017). Advanced renal osteodystrophy includes orofacial disorders such as decreased trabeculation, ground-glass appearance of bone, bone demineralization, radiolucent fibrocystic lesions, abnormal bone healing, decreased thickness of cortical bone, lytic areas of bone, jaw fracture, radiolucent giant cell lesions, and metastatic soft-tissue calcifications which have been reported in the literature (Yuan et al., 2017). It is caused by hyperparathyroidism, although becoming less common, and can affect the mandible, cause brown tumors, expand the skeletal bases, and greater tooth movement (Summers et al., 2007).

#### 3.5.8 Brown tumor

A brown tumor is a severe form of hyper-parathyroid bone disease found in patients with end-stage renal disease (Spasovski et al., 2009). It may affect up to 54 % of those on hemodialysis, according to the International Burden of Chronic Kidney Disease and Secondary Hyperparathyroidism (Shavlokhova et al., 2021). It is a localized bone lesion produced by enhanced osteoclastic activity and fibroblast proliferation (Queiroz et al., 2016). The term “brown tumor” refers to an accumulation of hemosiderin pigment, which gives the lesion a brown appearance under the microscope (Shavlokhova et al., 2021). Within the craniofacial, there are reports of tumors involving the maxilla, palatine bone, temporal, nasal, orbit, and paranasal sinuses. These tumors are more prevalent in the mandible than in the maxilla and are three times more common in women (over 50 years old) than in men (Queiroz et al., 2016).

#### 3.5.9 Gingival hyperplasia

One of the most common oral manifestations of renal disease is gingival enlargement. It mostly involves the labial, interdental papillae; however, more severe cases may also involve the gingival margins at the lingual and palate. Drugs frequently cause gingiva hypertrophy, which is now typically linked to cyclosporin use or calcium channel blockers (Proctor et al., 2005, Summers et al., 2007). Intake of nifedipine in the presence of dental plaque can cause gingival enlargement (Khan et al., 2017). Good dental hygiene should be performed in patients with gingival hyperplasia since it may help the condition independently. There may be benefits to using chlorhexidine mouthwash (Summers et al., 2007).

#### 3.5.10 Hairy leukoplakia

A white adherent patch or plaque known as leukoplakia can develop on the oral mucosa. A type of leukoplakia known as oral hairy leukoplakia manifests as a white, hairy-appearing lesion on one or both lateral borders of the tongue that is unable to wipe using a scraper (Levarda-Hudolin, 2016). Additionally, buccal, labial, or palatal mucosa may be affected. Most patients have impaired immune systems related to this condition (Levarda-Hudolin, 2016). Most occurrences of hairy leukoplakia do not require treatment because it is typically asymptomatic and has no chance of becoming cancerous. However, although recurrence is common so as long as the cause of immunosuppression is there, antiviral medications like acyclovir or ganciclovir, topical podophyllin, or even surgical excision might be performed (Levarda-Hudolin, 2016).

#### 3.5.11 Neoplasm

The most frequent oral *peri*-oral tumors in these patients are lip cancer, followed by Kaposi sarcoma (López-Pintor et al., 2011). Lip cancer has been reported in these patients to range from 5 % to 22.9 % (López-Pintor et al., 2011). Squamous cell carcinoma of the lower lip was reported in renal transplant patients to be higher than in the normal population (López-Pintor et al., 2011). Interaction of several factors is associated with the increased risk of the development of cancer in renal transplant patients; these include chronic previous uremic state and cumulative exposure to immunosuppression that eventually leads to carcinogenic effects (López-Pintor et al., 2011). Compared to the general population, kidney transplant recipients have a much higher frequency of malignant lesions, and the risk rises each year following the transplant; therefore, it is essential to be aware that Kaposi sarcoma might mimic gingival hyperplasia when it is presented orally (Levarda-Hudolin, 2016). Although Kaposi sarcoma has been closely linked to immunosuppression and the human herpes virus 8, the exact cause of the disease is unclear (Levarda-Hudolin, 2016).

### 3.6 Patient assessment

All renal transplant patients must go through comprehensive history taking and complete examination of the lip and intraoral mucosa after renal transplantation to detect the presence and monitor the progression of renal osteodystrophy or any abnormalities through regular dental visits (Bodnar et al., 2014, ES, S., Kumar, D., Raghuveer, H. P., NT, P., & Rangan, V. , 2014, López-Pintor et al., 2011). Dental clinicians should carefully evaluate the oral findings and general condition of the renal disease patient (Bodnar et al., 2014, Yuan et al., 2017). Discussion with the patient’s nephrologist is essential to gather all the data, including risk factors, drug excretion or metabolism, surgery preferred time, degree of CKD, ongoing therapy, past and current treatments, causes, and clinical features (Yuan et al., 2017). History of any systematic illness should be recorded, including diabetes, abnormal hemostasis, immune status, bone involvement, anemia, and cardiovascular disease. Routine HBV, HCV, and HIV antibody serology should be considered (Bodnar et al.,; ES et al., 2014;2014Yuan et al., 2017). Blood tests and residual bone evaluation are also essential for patients with renal failure (Yuan et al., 2017).

### 3.7 Treatment plan modification

Dental treatment in patients with renal failure is considered a critical challenge because of the complications associated with chronic kidney disease. These complications include bleeding, infection, and a decrease in glomerular filtration rate, where drug metabolism will be altered, and modification of the drug used should be considered with renal patients and if any bony lesions are present (Borawski et al., 2006, Nadeem et al., 2009, Yuan et al., 2017). The decision of antibiotic prophylaxis should be discussed with the patient's nephrologist regarding recent lab results (Borawski et al., 2006, Craig, 2007, ES, S., Kumar, D., Raghuveer, H. P., NT, P., & Rangan, V. , 2014). It is necessary to avoid recording blood pressure measurements or drug injections into the arm with vascular access (Es et al., 2014). The most common oral findings for pediatric patients include brown teeth discoloration, delayed eruption of permanent teeth, and tooth structure abnormalities. Calcification or constricting of the pulp chamber might occur in children and adults (Al Nowaiser et al., 2003, Yuan et al., 2017). Low caries risk is reported in pediatric patients with renal disease because of the highly buffered and alkaline saliva that results from elevated phosphate and urea concentrations (Al Nowaiser et al., 2003, Yuan et al., 2017). Severe erosions, tooth mobility, crowding, and malocclusion can be presented in chronic kidney disease patients. Patients on hemodialysis eventually have a greater risk of tooth loss than normal patients (Yuan et al., 2017). Dental surgery should be done on the day following hemodialysis to eliminate all circulating toxins (ES, S., Kumar, D., Raghuveer, H. P., NT, P., & Rangan, V. , 2014, Yuan et al., 2017).

### 3.8 Risk of bleeding

Patients with end-stages and on dialysis are supposed to have a risk of hemorrhage due to dysfunction of platelet due to anti-coagulation medications (Heparin), which is used to facilitate the dialysis procedure; thus, hemodialysis patients should avoid any dental procedure on the first day, and the best way is to consult with the nephrologist before starting any procedure (Klassen and Krasko, 2002, Georgakopoulou et al., 2011, Gupta et al., 2015, Raja and Coletti, 2006, Yuan et al., 2017). Anticoagulant usage during hemodialysis and maintaining vascular access can cause bleeding in CRF patients (Es et al., 2014). Patients with lower platelet counts, lower platelet adhesiveness, higher prostacyclin activity, lower availability of platelet factor, and more capillary fragility will all result in greater blood loss. Anemia also can exacerbate bleeding (Es et al., 2014). There have also been reports of petechial and ecchymosis-like lesions, gingival hemorrhages, and ulcerations. Anti-fibrinolytic drugs, fresh frozen plasma, vitamin K, and platelet replacement may be administered for patients with significantly prolonged bleeding or clotting times (Es et al., 2014). Invasive oral procedures may require electrocautery to stop bleeding (Es et al., 2014). These patients may have anemia, mostly caused by decreased erythropoietin secretion and other factors, and increased susceptibility to bleeding because of aggravated hemolysis (Bodnar et al., 2014). Anemia is associated with platelet dysfunction in some patients, and this problem is fully corrected once transplantation is done (Eigner et al., 1986). CKD patients have platelet dysfunction, increasing the tendency to prolong bleeding (Almeida et al., 2016). The average hematocrit of dialysis patients is approximately 25 % (Eigner et al., 1986).

### 3.9 Risk of infection

The renal transplant or hemodialysis patient is highly susceptible to infection, particularly immediately after surgery (Yuan et al., 2017). Any patients who consume an immunosuppression medication course are at risk of candida infection; these include pseudomembranous candidiasis and chronic atrophic candidiasis (Klassen and Krasko, 2002, Yuan et al., 2017). In addition, the viral risk of infection is approximately 50 % in patients that are positive for herpes simplex (Klassen & Krasko, 2002). However, these days the use of appreciating antiherpetic viral drugs can significantly reduce the risk of infection by taking into consideration the long period of post-allograft immunosuppression that can lead to human herpesvirus 8 conjunction with Kaposi’s sarcoma with the susceptibility of hepatitis B or C being higher in patients on dialysis (Georgakopoulou et al., 2011, Klassen and Krasko, 2002). Most transplant patients are on corticosteroids and immunosuppressive, and that is why kidney transplant candidates must have an adequate dental examination and dental treatment pre-transplant and periodic follow-up post-transplant, as any source of infection can flare up and cause a widespread of local and systemic infection (Saif et al., 2011a, Saif et al., 2011b). Immunosuppressive medications, which are required for kidney transplants, have several short and long-term side effects that can be fatal to the patient, including infection, elevated cardiovascular risks, and neoplastic diseases (Almeida et al., 2016, López-Pintor et al., 2010). The cell-mediated immune response is suppressed by immunosuppressive therapy (López-Pintor et al., 2010). This means a greater risk of oral infection and many other associated complications for dentists. Because oral pathogens cannot be suppressed and destroyed by the immune system in immunosuppressed patients, they are more likely to cause local damage and opportunistic infections (López-Pintor et al., 2010). During immunosuppressive medication, oral lesions may also appear due to adverse effects and drug interactions (López-Pintor et al., 2010). Patients with long-term dialysis or renal transplants are often subjected to tooth loss and need further use of dental prostheses (Osiak et al., 2020). However, dental prostheses can be colonized by bacteria and, therefore, cause several pathologies in the mucous membranes of the oral cavity; these kinds of pathologies can be very difficult to treat (Osiak et al., 2020). The occurrence of pathologic lesions in the oral cavity in patients after the transplant of vascularized organs may result from long-term pharmacologic immunosuppression, which inhibits the immune response and thus increases the risk of infection in the oral cavity as well as of other diseases (Osiak et al., 2020). CKD patients can also be presented with infective endocarditis (Yuan et al., 2017).

### 3.10 Drug-drug interaction and metabolism

Several drugs can be excreted by the renal system, resulting in reduced renal function (Yuan et al., 2017). Renal dysfunction affects how certain medications are metabolized and eliminated; dose modification or frequency change is required (Singh et al., 2014). Due to their nephrotoxicity, tetracyclines and aminoglycoside antibiotics should not be prescribed. The preferred antibiotics are penicillin, clindamycin, and cephalosporins, which can be given at standard doses (Singh et al., 2014). Regarding non-narcotic analgesics, paracetamol is the drug of choice for episodic pain. Aspirin has anti-platelet activity; thus, uremic people should avoid using it (Singh et al., 2014). In the more severe stages of renal failure, it is advised to reduce or entirely avoid the dose of the other nonsteroidal anti-inflammatory medications because they suppress prostaglandins and have a hypertensive effect (Singh et al., 2014). Without the requirement for dose modifications, benzodiazepines can be prescribed, while severe sedation is possible. Since the liver metabolizes narcotic analgesics, dose adjustments are typically unnecessary (Singh et al., 2014). Medications that can cause nitrogen retention, such as tetracycline, nephrotoxic drugs such as aminoglycosides, cephalosporins, aspirin, and nonsteroidal anti-inflammatory drugs that can exacerbate gastrointestinal irritation and bleeding, should be avoided, particularly in patients on dialysis, and the doses of drugs should be adjusted according to the nephrologist’s recommendations (Bodnar et al., 2014). Anti-fungal (azole) should be avoided in renal transplant patients due to increased serum ciclosporin and tacrolimus (Saif et al., 2011a, Saif et al., 2011b). Dentists should avoid multiple unnecessary drug prescriptions for patients with renal disease and extend the interval between doses depending on the degree of elimination (Yuan et al., 2017). Nephrotoxic drugs should be completely avoided (Yuan et al., 2017). Oral lesions known as mTOR-inhibitor-associated stomatitis which develops during therapy with mammalian target of rapamycin inhibitors (mTORI) (Calvo et al., 2019). As an immunosuppressive medication to prevent rejection following a kidney transplant, mTORI rapamycin is approved by the US Food and Drug Administration (FDA) (Meiller et al., 2015). The oral lesions linked to mTORI toxicity are not the same as the well-known mucositis caused by radiation and chemotherapy, they frequently have a diameter of less than 0.5 cm, occur as multiple or a single ulceration, and heal in about two weeks (Meiller et al., 2015). Dose adjustment for patients on dialyzes is illustrated in Table 1.

**Table 1:** Dose adjustment for patients on dialysis (Yuan et al., 2017).

Drug	Common dose	Adjustment
Amoxicillin	250–500 mg every 8 h	Prolongation of the dosing interval every 24 h
Doxycycline	–	No adjustment needed
Erythromycin	–	No adjustment needed
Tetracycline	250–500 mg two to four times daily	Prolongation of the dosing interval every 24 h
Clindamycin	–	No adjustment needed
Ampicillin	1–2 g ampicillin and 0.5–1 g sulbactam every 6–8 h	Prolongation of the dosing interval every 12–24 h
Acyclovir	200–800 mg every 4–12 h	Prolongation of the dosing interval 200 mg every 12 h
Ketoconazole	–	No adjustment needed
Aspirin	Avoid	–
Ibuprofen	Avoid	–
Diclofenac	Avoid	–
Paracetamol	300–600 mg every 4 h	Prolongation of the dosing interval every 8–12 h

### 3.11 Emergency

In any emergency, the patients are considered a suitable source of data collection due to their need to monitor their signs and symptoms (Sowell, 1982). These patients are usually well-informed regarding their condition (Sowell, 1982). A patient receiving infrequent hemodialysis has a higher risk of infection, a tendency to bleed because of insufficient platelet factor III activity, a potential for high blood pressure, a tendency for osteodystrophy and its physiological impacts, a compromised stress response because of steroid therapy, and the risks associated with a high prevalence of subclinical viral hepatitis. Immunosuppressive medication therapy further decreases the host resistance to infection in the kidney transplant patient. If these patients are to avoid issues related to bacteremia of oral origin, preventive measures must be employed in preoperative and postoperative dental care (Bottomley et al., 1972). In case of an invasive dental procedure patient with a significantly increased bleeding tendency or receiving blood thinners, drugs may be prescribed, or electrocautery used to control the bleeding (Gupta et al., 2015). If a patient with stage 1 or 2 renal disease attends the dental clinic because of pain, we can treat the patient normally. However, for stages 3, 4, and 5, or patients on dialysis, a consultation should be taken from the renal physician; in addition, help can be asked from special care dentistry (Saif et al., 2011a, Saif et al., 2011b). If a patient has swelling or sepsis, drainage of the pus through the tooth should be obtained, and the antibiotic dose should be adjusted. In case the patient needs an elective extirpation of the pulpal tissue, it should be delayed until consultation is received from the renal physician (Saif et al., 2011). If any dental emergency occurs after renal transplant surgery or if the body rejects the kidney transplant while the patient receives an immunosuppression course, the dental emergency treatment should be done in a hospital. Any planned dental procedure should be consulted with the nephrologist first (Georgakopoulou et al., 2011).

### 3.12 Patient capability

The treatment for patients suffering from CKD should be performed on several levels, considering the high risk of infection, the tendency to bleed, and the impaired ability to eliminate medications (Bodnar et al., 2014). It was reported by many authors that there is a significant poor oral hygiene measurement among patients with advanced stages of chronic kidney disease. In addition, studies showed that patients on dialysis brush their teeth once or more every day (Yuan et al., 2017). Yet, few of them use floss and demonstrate infrequent dental visits. Patients on dialysis also require an obvious need for dental treatment (Yuan et al., 2017). All patients with chronic kidney disease will benefit from dental care; thus, they should be routinely checked for oral lesions and treated as necessary. This is because the oral aspect of CKD impacts the patient's quality of life (Oyetola et al., 2015). Similarly, every candidate for a kidney transplant should receive a dental examination, proper dental care, and ongoing monitoring after the transplant. In order to manage chronic renal patients and provide the highest possible quality of life, nephrologists and dentists need to work more closely together (Oyetola et al., 2015).

### 3.13 For patients under conservative care, the following should be considered

• Physician consultation about physical status and control level.
• Dental treatment should be avoided if the disease is unstable.
• Screening for the bleeding disorder before surgery, including hemoglobin, bleeding time, hematocrit, and platelet count.
• Blood pressure should be monitored closely.
• Good surgical technique should be considered.
• Nephrotoxic drugs should be avoided, including aspirin, acetaminophen, NSAID, and acyclovir.
• Dosage adjustment of drugs metabolized by the kidney.
• Management of orofacial infections is a must with sensitivity and culture tests and antibiotics.
• Hospitalization should be considered for major procedures or severe infection
• Corticosteroid supplementation, as indicated**.** (Little et al., 2018).

### 3.14 For patients receiving hemodialysis, recommendations are the same as conservative care. In addition to the following:

• Awareness of arteriovenous shunt.
• Consultation with the physician regarding the risk of infective endarteritis.
• Blood pressure cuff and IV medications should be avoided in the arm with a shunt.
• Dental care should be avoided on the day of treatment (prefer the day after the hemodialysis).
• Antimicrobial prophylaxis should be considered.
• Corticosteroid as indicated.
• Liver function should be assessed. (Little et al., 2018).

## 4 Conclusion

Patients with varying degrees of renal disease could be seen in the dental chair; thus, every dentist should be well-informed regarding dental considerations and oral manifestations when treating a patient with any renal disease. Simple treatment plan modification can prevent the evolution of mild to critical medical or dental complications in renal disease patients.

Ethical approval

This article does not include any studies involving human participants or animals performed by the author. An exemption letter was provided by the Institutional Review Board (IRB) (No. E-23–8090).

## CRediT authorship contribution statement

**Hamad Albagieh:** Conceptualization, Project administration, Supervision, Validation. **Asim Alosimi:** Resources, Writing – review & editing, Writing – original draft. **Abdulrahman Aldhuhayan:** Resources, Writing – review & editing. **Abdulrahman AlAbdulkarim:** Resources, Writing – review & editing. **Bader Fatani:** Resources, Writing – review & editing. **Abdulaziz Alabood:** Resources, Writing – review & editing.

## Declaration of competing interest

The authors declare that they have no known competing financial interests or personal relationships that could have appeared to influence the work reported in this paper.
